# Impaired Thymic Output Can Be Related to the Low Immune Reconstitution and T Cell Repertoire Disturbances in Relapsing Visceral Leishmaniasis Associated HIV/AIDS Patients

**DOI:** 10.3389/fimmu.2020.00953

**Published:** 2020-05-20

**Authors:** Maria Luciana Silva-Freitas, Gabriela Corrêa-Castro, Glaucia Fernandes Cota, Carmem Giacoia-Gripp, Ana Rabello, Juliana Teixeira Dutra, Zilton Farias Meira de Vasconcelos, Wilson Savino, Alda Maria Da-Cruz, Joanna Reis Santos-Oliveira

**Affiliations:** ^1^Laboratório Interdisciplinar de Pesquisas Médicas, Instituto Oswaldo Cruz, Fundação Oswaldo Cruz (FIOCRUZ), Rio de Janeiro, Brazil; ^2^Núcleo de Ciências Biomédicas Aplicadas, Instituto Federal de Educação, Ciência e Tecnologia Do Rio de Janeiro (IFRJ), Rio de Janeiro, Brazil; ^3^Centro de Referência em Leishmanioses, Instituto René Rachou, Fundação Oswaldo Cruz (FIOCRUZ), Belo Horizonte, Brazil; ^4^Laboratório de AIDS e Imunologia Molecular, Instituto Oswaldo Cruz, Fundação Oswaldo Cruz (FIOCRUZ), Rio de Janeiro, Brazil; ^5^Laboratório de Alta Complexidade, Instituto Nacional de Saúde da Mulher, da Criança e Do Adolescente Fernandes Figueira (IFF), Fundação Oswaldo Cruz (FIOCRUZ), Rio de Janeiro, Brazil; ^6^Laboratory on Thymus Research, Oswaldo Cruz Institute, Oswaldo Cruz Foundation (FIOCRUZ), Rio de Janeiro, Brazil; ^7^National Institute of Science and Technology on Neuroimmunomodulation, Oswaldo Cruz Institute, Oswaldo Cruz Foundation (FIOCRUZ), Rio de Janeiro, Brazil; ^8^Rede de Pesquisas em Saúde Do Estado Do Rio de Janeiro/FAPERJ, Rio de Janeiro, Brazil; ^9^Disciplina de Parasitologia/DMIP, Faculdade de Ciências Médicas, Universidade do Estado do Rio de Janeiro (UERJ), Rio de Janeiro, Brazil

**Keywords:** visceral leishmaniasis/HIV-1 co-infection, thymic output, TCRVβ repertoire, relapses, immune response

## Abstract

**Background:** Visceral leishmaniasis/HIV-co-infected patients (VL/HIV) accounts for around 8% of VL reported cases in Brazil. Relapses of *Leishmania* infection after anti-leishmanial treatment constitute a great challenge in the clinical practice because of the disease severity and drug resistance. We have shown that non-relapsing-VL/HIV (NR-) evolved with increase of CD4^+^ T-cell counts and reduction of activated CD4^+^ and CD8^+^ T cells after anti-leishmanial treatment. This immune profile was not observed in relapsing-VL/HIV patients (R-), indicating a more severe immunological compromising degree. Elevated activation status may be related to a deficient immune reconstitution and could help to explain the frequent relapses in VL/HIV co-infection. Our aim was to evaluate if this gain of T cells was related to changes in the peripheral TCRVβ repertoire and inflammatory status, as well as the possible thymus involvement in the replenishment of these newly formed T lymphocytes.

**Methods:** VL/HIV patients, grouped into non-relapsing (NR- = 6) and relapsing (R- = 12) were evaluated from the active phase up to 12 months post-treatment (mpt). HIV-infected patients (non-VL) and healthy subjects (HS) were included. The TCRVβ repertoire was evaluated *ex vivo* by flow cytometry, whereas the plasmatic cytokine levels were assessed by Luminex assay. To evaluate the thymic output, DNA was extracted from PBMCs for TCR rearrangement excision circles (TREC) quantification by qPCR.

**Results:** VL/HIV cases presented an altered mobilization profile (expansions or retractions) of the TCRVβ families when compared to HS independent of the follow-up phase (*p* < 0.05). TCRVβ repertoire on CD4^+^ T-cells was more homogeneous in the NR-VL/HIV cases, but heterogeneous on CD8^+^ T-cells, since different Vβ-families were mobilized. NR-VL/HIV had the inflammatory pattern reduced after 6 mpt. Importantly, VL/HIV patients showed number of TREC copies lower than controls during all follow-up. An increase of recent thymic emigrants was observed in NR-VL/HIV individuals at 10 mpt compared to R- patients (*p* < 0.01), who maintained lower TREC contents than the HIV controls.

**Conclusions:** VL/HIV patients that maintain the thymic function, thus generating new T-cells, seem able to replenish the T lymphocyte compartment with effector cells, then enabling parasite control.

## Introduction

Visceral leishmaniasis (VL) is a neglected tropical disease associated with poverty, being a public health issue in endemic countries, mainly in tropical and subtropical regions (1, 2). Most of VL cases in the Americas occurs in Brazil, where around 4,000 new cases are reported annually (1–3). An increasing number of HIV-associated VL (VL/HIV) cases has been identified since 2001 reaching 7.8% of the whole VL cases reported in 2017 (3). Noteworthy, VL/HIV patients frequently fail to respond successfully to treatment, exhibiting a high rate of drug toxicity, relapses and mortality (1, 2, 4).

VL/HIV patients evolve with an intense immunosuppression and, paradoxically, potentiated cellular activation, despite antiretroviral therapy (ART) and clinical remission of VL (2, 5). We previously demonstrated that *Leishmania* infection was the main co-factor associated with the immune activation state in HIV-infected individuals (5). Allied to this, elevated levels of lipopolysaccharide (LPS) pointed out that microbial products from the gut lumen translocation, could also be involved in the exacerbated pro-inflammatory status of VL alone (6) and VL/HIV co-infected patients (2, 7). Plasma inflammatory cytokines levels, as well as soluble molecules associated with inflammation such as d-dimers, neopterin, soluble CD163 and leptin levels have been described as important predictors of severity and death in VL (8–12).

We showed that relapsing-VL/HIV patients maintained low CD4^+^ T lymphocyte counts, higher percentages of activated CD4^+^ and CD8^+^ T cells, sCD14 and anti-*Leishmania* IgG3, even 12 months after anti-leishmanial treatment, whereas this immune profile was reverted in non-relapsing-VL/HIV patients after anti-*Leishmania* treatment (13). This indicates intense activation leading to an exhaustion of the immune response to the parasite, which may contribute to the frequent VL relapses or even faster disease progression (2, 14).

Continuous cellular activation induces immunosenescence and exhaustion of primary immune resources. As a consequence, decreased generation of new T cells and lower peripheral T-cell repertoire diversity takes place (15–17). In this respect, it is relevant to point out that the thymus of HIV patients is also affected, compromising both the lymphoid and microenvironmental compartments of the organ (18). Thymic capacity of exporting mature T lymphocytes can be ascertained by quantifying the T-cell receptor excision circles (TRECs). These circles are generated intrathymically during the somatic gene rearrangement process that generates the T-cell receptor (TCR) and are unique of naive Tαβ cells, allowing the identification in the periphery of the so-called recent thymic emigrants (RTEs) (19–21).

In a second vein, HIV-positive patients evolve with disturbances in the generation of the T lymphocyte receptor Vβ repertoire (TCRVβ) (17), which can potentially compromise the effector responsiveness to a variety of antigens, including *Leishmania*. In this context, disorders of the TCRVβ repertoire have been related to the immunopathogenesis of several diseases, such as cancer (22); rheumatoid arthritis (23); hematological comorbidities (24); Chagas disease (25); cutaneous leishmaniasis (26, 27); and HIV/AIDS (28, 29). HIV-positive patients with a restrict mono-oligoclonal TCRVβ repertoire profile present a rapid AIDS-progression, suggesting that the T-cell repertoire disturbances do influence the HIV/AIDS prognosis (28) and of its association with other infections, such as Epstein Barr virus (30). Moreover, after ART, a change in the profile of the TCRVβ repertoire is observed since, not only newly Vβ families appear in the periphery, but also others are positive or negatively mobilized (29, 31).

We recently showed that VL-HIV co-infected patients with satisfactory clinical evolution and no recurrence of VL (non-relapsing) presented increased content of the circulating CD4^+^ T cell pool, indicating that they still have the ability to replenish the peripheral T cell compartment. This was not observed in relapsing VL-HIV patients, suggesting a sort of burnout of T cell sources (32). The main source of these T cells, peripheral lymphoid organs or thymus, can be affected by both *Leishmania* and HIV infection (18, 33, 34). Accordingly, it is conceivable that the compromising degree of one of these compartments may be related to a deficient immune response. These features may contribute to the lack of parasite control which in turn could explain the frequent relapses in VL/HIV (13, 14). Thus, the aim of this study was to evaluate if the gain of T cells observed in NR-VL/HIV patients after anti-*Leishmania* treatment (13) was related to changes in the mobilization profile of the peripheral TCRVβ repertoire, and whether the thymus is involved in the replenishment of newly T cells, especially in non-relapsing VL/HIV co-infected patients.

## Materials and Methods

### Casuistic of the Study and Ethical Aspects

Eighteen VL/HIV co-infected patients were recruited from Hospital Eduardo de Menezes, Belo Horizonte, Brazil, being prospectively followed from February 2011 up to March 2013. The same patient cohort was previous evaluated by Silva-Freitas et al. (13) and those studies revealed differences in cellular activation and a senescent phenotype. Briefly, these patients were grouped in relapsing VL/HIV (R-VL/HIV; *n* = 12) e non-relapsing VL/HIV (NR-VL/HIV; *n* = 6) respectively, according to the occurrence or not of VL relapse episodes throughout life. Clinical aspects, diagnosis, treatment and ethical aspects were previously reported (13). Parasitological tests (direct exam and culture) from bone marrow aspirates were used to confirm the VL diagnosis in all patients. At that time, the first line treatment recommended by the Brazilian Ministry of Health for VL/HIV patients was amphotericin B deoxycholate for 4 weeks (35). After treatment, secondary prophylaxis with amphotericin B was offered every 2 weeks to those VL/HIV patients that maintained absolute CD4^+^ T lymphocyte counts below 350 cells/mm^3^ (36).

The immunological parameters were evaluated in the following monitoring periods: active phase, early post-treatment, 6 and 10 (for TREC assessment) and 12 months post-treatment (mpt). Patients infected only with HIV as well as healthy subjects (HS) were included as controls. This study was approved by ethical review boards of Hospital Eduardo de Menezes and of the Oswaldo Cruz Foundation (René Rachou and Oswaldo Cruz Institutes).

### Evaluation of TCRVβ Repertoire Levels by Flow Cytometry

To evaluate the Vβ repertoire of T lymphocytes we used the IOTest®Beta Mark kit (Beckman-Coulter, Fullerton, CA, EUA), which includes specific monoclonal antibodies for 24 Vβ chains belonging to 19 out of 26 Vβ families known. The following 24 Vβ families were evaluated: Vβ1, 2, 3, 4, 5.1, 5.2, 5.3, 7.1, 7.2, 8, 9, 11, 12, 13.1, 13.2, 13.6, 14, 16, 17, 18, 20, 21.3, 22, 23. In this kit, three Vβ different chains were simultaneously analyzed in a single dotplot, being labeled with the following fluorochromes: FITC or PE or with the FITC-PE combination. Therefore, this flow cytometry protocol allowed to analyze the expression of Vβ chains in the subpopulations of CD4^+^ and CD8^+^ T lymphocytes using anti-CD4 PercP (Peridinin-chlorophyll proteins) and anti-CD8 APC (allophycocyanin) monoclonal antibodies. The gate of these subpopulations was defined within the CD3^+^ T lymphocyte compartment, in a tube containing anti-CD4 PercP, anti-CD8 APC and anti-CD3 FITC monoclonal antibodies. From this lymphocyte subpopulations (CD4^+^ or CD8^+^ T cells), a dotplot was created to define the percentages of cells expressing such Vβ family. Peripheral blood mononuclear cells (PBMCs) obtained of the Ficoll-Hypaque gradient centrifugation were used to *ex vivo* immunophenotyping and all samples were acquired by a FACSCalibur® device (BD Biosciences, San Jose, CA, USA). For each sample, 20,000 events were acquired within the lymphocyte gate. The cytometry analyses were performed using the Cell Quest Pro^TM^ software (BD Biosciences, San Jose, CA, USA).

### Quantification of Cytokine Levels

A multiplex assay was performed to quantify the serum levels of the following cytokines: IFN-γ, TNF, IL-1β, IL-2, IL-4, IL-5, IL-6, IL-7, IL-8, IL-10, IL-12, IL-13, IL-17, MCP-1, and MIP-1β. Cytokine contents were calculated by Luminex technology (Bio-Plex Workstation; Bio-Rad Laboratories, USA). Data analysis was performed using the software provided by the manufacturer (Bio-Rad Laboratories, USA). Recombinant cytokines were used to establish standard curves and the sensitivity of the assay. Results were expressed as Median Fluorescence Intensity (MFI) (37). The MFI of the last point of each standard curve was used to determine the detection limit of each cytokine.

### Quantification of T Cell Receptor Excision Circles (TRECs) by qPCR

PBMCs previously cryopreserved were thawed and the DNA was extracted directly from the cells using the QIAamp DNA Blood Mini kit, following the manufacturer's instructions (Qiagen, Manchester, UK). The DNA was extracted of an initial concentration of cells which ranged from 1 to 5 million PBMC/mL. After extraction, the eluted DNA was quantified through of NanoDrop 2000c spectrophotometer (Thermo Fisher Scientific, Wilmington, USA). TRECs were quantified by real-time quantitative Polymerase Chain Reaction (qPCR), according to the principles of the test established by Douek et al. (19). Briefly, 2 μL of extracted DNA was added in MicroAmp® Optical 96-well reaction plate (Applied Biosystems®) with 3 μL of TREC reaction mix, which consisted in: miliQ water; TREC primers: Forward (5′-CAC ATC CCT TTC AAC CAT GCT-3′) and Reverse (5′-GCC AGC TGC AGG GTT TAG G-3′); probe TREC (6-FAM-ACA CCT CTG GTT TTT GTA AAG GTG CCC ACT-39-TAMRA); and the 2x TaqMan Universal Master Mix II enzyme (Applied Biosystems®). Reaction final volume was 5 μL/well. To perform TREC quantification, a plasmid containing TREC sequence cloned, kindly provided by Dr. Daniel Douek (Vaccine Research Center, NIH, USA) was serially diluted from 10^6^ to 10^−1^ TREC copies/μL and used as standard curve amplified in parallel in each experiment. To control the quality of the assay and the integrity of the extracted DNA, the RNAseP endogenous constitutive gene was quantified in all samples. Moreover, in order to normalize the copy number of the target gene (TREC/μL) by the number of total cells contained in each sample, we used a standard curve of RNAseP that was serially diluted from 10^6^ up to 10^1^ PBMC/μL. Each sample was run in duplicate and the qPCR assay was performed in the 7,500 Real-Time PCR System equipment (Life Technologies). The analysis was performed in the 7,500 software v2.3 (Life Technologies) and the final result was expressed by 10^6^ cells, as TREC copies/10^6^PBMC.

### Statistical Analysis

The statistical analyses were performed using GraphPad Prism software (version 6.0, San Diego, CA, USA). For comparisons between VL/HIV co-infected patients and control groups, we used non-parametric tests: Mann Whitney when two groups were analyzed; ANOVA (Kruskal–Wallis) and Dunns post-test when three or more groups were simultaneously compared. Parametric test (Wilcoxon) was applied when the same patient was compared in his/her different phases of follow-up. Differences were considered statistically significant when the *p* value was ≤0.05. Heatmap analyses were performed to evaluate the differential expression patterns of TCRVβ repertoire on T cell subpopulations in VL/HIV patients. For this, the online software Heat mapper® (Wishart Research Group at the University of Alberta) was applied to draw a heatmap from a spreadsheet containing the expression index of each TCRVβ family for each patient evaluated, using the following formula: % Y-Vβ family expression of the X-patient in a given follow-up period divided by average of the % Y-Vβ family expression in the healthy controls. The clustering method used for analysis was the average linkage, and the distance measurement method applied was Euclidean.

## Results

### Mobilization of the CD4^+^ and CD8^+^ T-Cell Vβ Repertoire After anti-*Leishmania* Treatment in Non-relapsing and Relapsing Visceral Leishmaniasis/HIV Co-infected Patients

As we previously demonstrated (13), NR-VL/HIV and R-VL/HIV patients presented very low levels of CD4^+^ T cells during active phase of VL (Table 1). However, at 6 mpt a significant increase in this population was observed in NR-VL/HIV patients but not in R-VL/HIV, remaining until 12 mpt. Although CD8^+^ T lymphocyte counts also increased in the NR-VL/HIV group after treatment, no significant differences were found throughout the follow-up when the patients were evaluated individually (Table 1). This indicates that the mobilization occurs mainly in the CD4^+^ T cell pool. Since NR-VL/HIV patients had a higher degree of immune reconstitution in comparison to R-VL/HIV cases, we investigated whether this T cell input was associated with newly TCRVβ repertoire diversity or even differentially mobilized.

**Table 1:** T cell absolute counts of non-relapsing and relapsing visceral leishmaniasis/HIV (VL/HIV) co-infected patients.

**VL/HIV group**	**Follow-up phase**	**CD4**^****+****^ **cells/mm**^****3****^ **Median [IQR]**		**CD8**^****+****^ **cells/mm**^****3****^ **Median [IQR]**		***n***
Non-relapsing	Active VL	86.5	[66.3–120.5]	364	[238.5–1,032]	6
	Early post-treatment	166.5	[120.3–263.8]	499	[386.8–961.8]	6
	6 mpt	297	[216.8–478.5]*	766.5	[428.8–1,872]	6
	12 mpt	350	[284.5–659.5]**	806	[651.5–1,408]	6
Relapsing	Active VL	116	[51.8–185]	418	[226.8–881]	12
	Early post-treatment	189.5	[96–257.8]	556	[390.3–1,305]	12
	6 mpt	132	[76–203]	425	[309–705]	11
	12 mpt	126	[39.25–232.3]	327.5	[218–1,476]	10

VL/HIV, patients with visceral leishmaniasis co-infected by HIV; mpt, months post-treatment; IQR, interquartile range; *Significant difference between non-relapsing (NR-) and relapsing (R-) VL/HIV patients at 6 mpt; **significant difference between non-relapsing (NR-) and relapsing (R-) VL/HIV patients at 12 mpt; n, number of patients in each follow-up phase.

**Figure 1:**
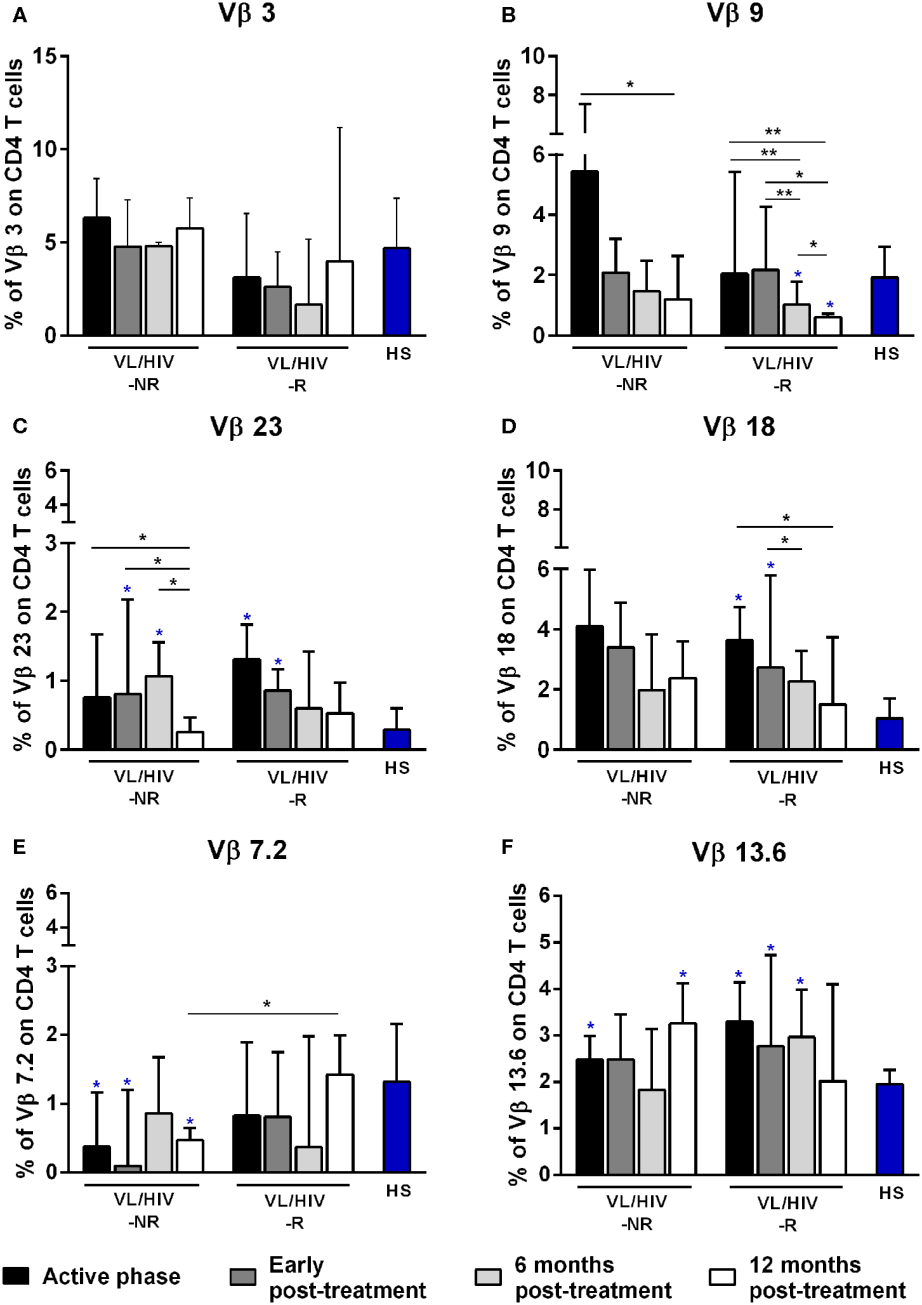
Percentages of the Vβ families on CD4^+^ T lymphocytes among non-relapsing (NR) and relapsing (R) visceral leishmaniasis/HIV (VL/HIV) co-infected patients. The mobilization percentages of Vβ3 **(A)**, Vβ9 **(B)**, Vβ23 **(C)**, Vβ18 **(D)**, Vβ7.2 **(E)**, and Vβ13.6 **(F)** in NR-VL/HIV co-infected patients in comparison to R-VL/HIV group. The blue asterisk represents the statistical differences in relation to healthy subjects (HS). The column bar represents the median values with interquartile range. *p < 0.05 **p < 0.005.

**Figure 2:**
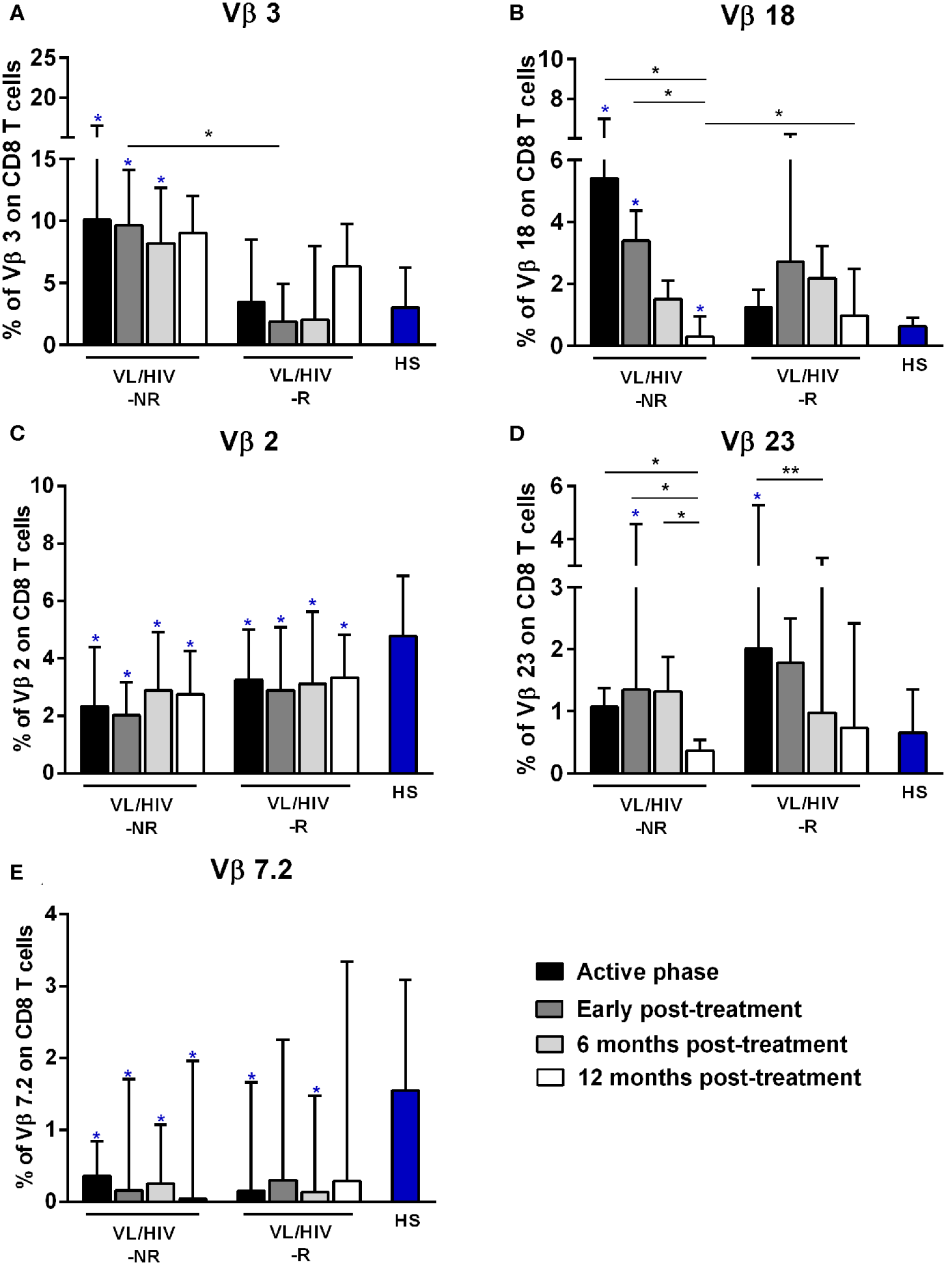
Percentages of the Vβ families on CD8^+^ T lymphocytes among non-relapsing (NR) and relapsing (R) visceral leishmaniasis/HIV (VL/HIV) co-infected patients. The mobilization percentages of Vβ3 **(A)**, Vβ18 **(B)**, Vβ2 **(C)**, Vβ23 **(D)**, and Vβ7.2 **(E)** in NR-VL/HIV co-infected patients in comparison to R-VL/HIV group. The blue asterisk represents the statistical differences in relation to healthy subjects (HS). The column bar represents the median values with interquartile range. *p < 0.05 **p < 0.005.

Then, we evaluated the overall TCRVβ mobilization profile of VL/HIV groups in comparison to HS. Considering both NR-VL/HIV and R-VL/HIV patients, in all clinical phases, the TCRVβ repertoire was significantly altered (higher or lower expression) in CD4^+^ T cells (70.8%; 17 out of 24 families) (Table 2) and in CD8^+^ T cells (50%, 12 out of 24 families) (Table 3). However, CD4^+^ T cells Vβ families were mobilized differently in NR-VL/HIV and R-VL/HIV groups (Table 2). In terms of CD8^+^ T cells, the NR-VL/HIV group maintained the expression of several families, whereas R-VL/HIV, not only mobilized few families but also their use was reduced in relation to healthy subjects. Nine families were simultaneously altered in both T cell subsets (Vβ18, Vβ5.1, Vβ20, Vβ5.2, Vβ23, Vβ11, Vβ14, Vβ4, Vβ7.2). Two out of the nine families showed a similar mobilization profile in the T cell subsets: Vβ23 and Vβ7.2 (Figures 1C,E, 2D,E). Interestingly, the Vβ7.2 family was significantly less expressed (below 1%) in both T cells in NR-VL/HIV and R-VL/HIV patients but in different time points of the follow-up (Figures 1E, 2E and Supplementary Figures 1A–D), suggesting a restriction of this family in VL/HIV patients.

**Table 2:** Differences in the CD4^+^ T-cell Vβ repertoire mobilization profile of non-relapsing and relapsing visceral leishmaniasis/HIV (VL/HIV) co-infected patients.

		**Non-relapsing (NR-VL/HIV)**				**Relapsing (R-VL/HIV)**			
		**Active**	**Early post-treat**	**6 mpt**	**12 mpt**	**Active**	**Early post-treat**	**6 mpt**	**12 mpt**
**Vβ** **families**	**5.3**								
	**7.1**								
	**3**								
	**9**								
	**17**								
	**16**								
	**18**								
	**5.1**								
	**20**								
	**13.1**								
	**13.6**								
	**8**								
	**5.2**								
	**2**								
	**12**								
	**23**								
	**1**								
	**21.3**								
	**11**								
	**22**								
	**14**								
	**13.2**								
	**4**								
	**7.2**								

* *Vβ families up-expressed (■) or down-expressed (■) in CD4^+^ T cells in comparison to healthy subjects. VL/HIV (patients with visceral leishmaniasis co-infected by HIV); Active (VL active phase); Early post-treat (immediately after anti-Leishmania post-treatment); 6 mpt (six months post-treatment); 12 mpt (12 months post-treatment); mpt (months post treatment)*.

**Table 3:** Differences in the CD8^+^ T-cell Vβ repertoire mobilization profile of non-relapsing and relapsing visceral leishmaniasis/HIV (VL/HIV) co-infected patients.

		**Non-relapsing (NR-VL/HIV)**				**Relapsing (R-VL/HIV)**			
		**Active**	**Early post-treat**	**6 mpt**	**12 mpt**	**Active**	**Early post-treat**	**6 mpt**	**12 mpt**
**Vβ** **families**	**5.3**								
	**7.1**								
	**3**								
	**9**								
	**17**								
	**16**								
	**18**								
	**5.1**								
	**20**								
	**13.1**								
	**13.6**								
	**8**								
	**5.2**								
	**2**								
	**12**								
	**23**								
	**1**								
	**21.3**								
	**11**								
	**22**								
	**14**								
	**13.2**								
	**4**								
	**7.2**								

* *Vβ families up-expressed (■) or down-expressed (■) in CD8^+^ T cells in comparison to healthy subjects. VL/HIV (patients with visceral leishmaniasis co-infected by HIV); Active (VL active phase); Early post-treat (immediately after anti-Leishmania post-treatment); 6 mpt (6 months post-treatment); 12 mpt (12 months post-treatment;) mpt (months post treatment)*.

A further analysis was to compare the TCRVβ mobilization in the active phase with the different phases after therapy. In relation to CD4^+^ T cells, three families were increased in NR-VL/HIV patients during the active phase, in comparison to HS: Vβ5.1, Vβ13.6 and Vβ1 but only Vβ5.1 and Vβ13.6 families remained significantly more expressed after treatment (Table 2; Figure 1F and Supplementary Figures 1A, 2). By contrast, among R-VL/HIV patients, six families were more elevated: Vβ18, Vβ13.6, Vβ23, Vβ22, Vβ13.2 e Vβ4 (Table 2; Figures 1C,D,F and Supplementary Figure 1A). All these families, except Vβ22, remained significantly higher immediately after anti-*Leishmania* treatment (Table 2; Figures 1C,D,F and Supplementary Figure 2). Four families displayed punctual changes in the TCRVβ mobilization: higher percentages of Vβ3 (throughout the follow-up; Figure 1A) and decreased expression levels of Vβ23 (12 mpt; Figure 1C) in NR-VL/HIV, decreased representation of Vβ18 in R-VL/HIV (12 mpt; Figure 1D) and decreased expression of Vβ9 in both groups (12 mpt; Figure 1B).

**Figure 3:**
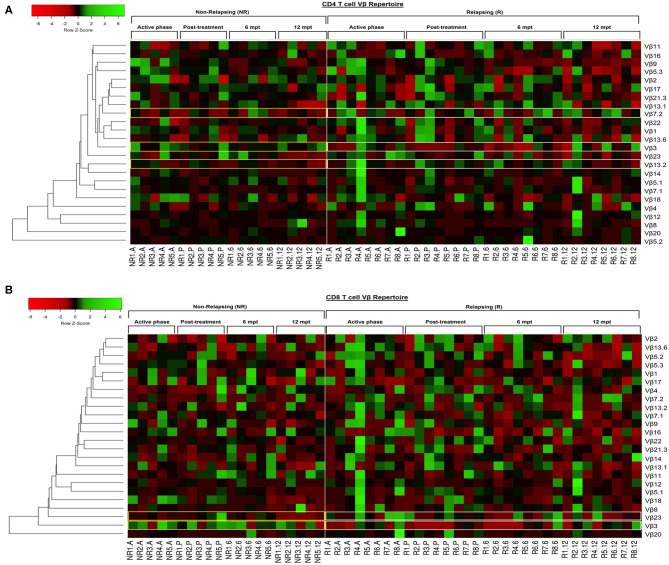
Individual pattern of Vβ repertoire mobilization on CD4^+^ and CD8^+^ T cells of the visceral leishmaniasis/HIV (VL/HIV) co infected patients during all the follow-up by heatmap analysis. To this analysis, the mobilization index was calculated so that the percentage of a given Vβ family presented by each VL/HIV patient was divided by mobilization mean of this Vβ family presented by healthy subjects. Each Vβ family is demonstrated in the line (*n* = 24) and each VL/HIV patient is represented in the column (NR- 1 to 5; R- 1 to 8) during all phases of follow-up (A, P, 6 and 12). The Vβ families were clustered in accordance with the similarity, using appropriate distance and clustering methods. The red and green scales represent a lower and higher mobilization index, respectively, of a given Vβ family on CD4^+^ T **(A)** and CD8^+^ T **(B)** cells for each VL/HIV patient seen individually. Active phase (A); Early post-treatment (P); 6 months post-treatment (6; 6 mpt); 12 months post-treatment (12; 12 mpt).

In order to provide an individual qualitative overview of the Vβ disturbances we used the heatmap strategy to represent the fold change of the percentages of each TCRVβ family presented by each VL/HIV patient in all phases of follow-up. The CD4^+^ TCRVβ repertoire analysis by heatmap confirmed that, although the Vβ9 family was highly mobilized during the active phase in NR-VL/HIV (4 out of 5 patients), it was reduced in both groups of co-infected patients after the anti-*Leishmania* treatment (Figures 1B, 3A; *p* < 0.05). The Vβ18 family also presented increased expression levels during the active phase and early after treatment against *Leishmania* among NR-VL/HIV patients (4 out of 5 patients), decreasing in the 6 and 12 mpt (Figure 3A), although such a decrease was not statistically significant (Figure 1B). This same pattern was observed in relation to Vβ22, Vβ23, and Vβ18 families, among the R-VL/HIV patients, where 5 out of 8 patients in the VL active phase mobilized these families (Figure 3A). At 12 mpt, the heatmap analysis confirmed the reduction in the expression levels of the Vβ13.1 (4 out of 5 patients) and Vβ23 families (all patients) among NR-VL/HIV group (Figure 3A). Also, it is important to mention Vβ3 family, whose mobilization tended to be higher among the majority of NR-VL/HIV patients throughout the follow-up (Figure 3A).

Concerning CD8^+^ T-cells, the Vβ18 and Vβ3 families were more mobilized (Vβ3 above 5%) in NR-VL/HIV patients during the active phase and after anti-*Leishmania* treatment when compared to HS (Table 3; Figures 2A,B and Supplementary Figure 1C). Although the R-VL/HIV group had lower percentages of Vβ3 family on CD8^+^ T cells in comparison to the NR- group, this family was the only one mobilized above 5% among R-VL/HIV patients at 12 mpt (Figure 2A and Supplementary Figure 1D). The representation of Vβ18 family on CD8^+^ T cells significantly decreased in NR-VL/HIV patients at 12 mpt in relation to active and post-treatment (Figure 2B), showing a different pattern of the R-VL/HIV group (Figure 2B). Among the R-VL/HIV group, only the Vβ23 on CD8^+^ T cells was more expressed during the VL active phase (Table 3; Figure 2D and Supplementary Figure 1A). In the NR-VL/HIV group, the mobilization percentages of this family significantly reduced at 12 mpt, in comparison to the previous phases (Figure 2D). Curiously, the percentages of CD8^+^ T cells expressing Vβ2 remained lower in both NR-VL/HIV and R-VL/HIV groups during all the follow-up period (Table 3; Figure 2C) in relation to HS, indicating a possible mobilization profile characteristic of the VL/HIV association in this subpopulation.

Noteworthy, practically no significant change in the TCRVβ mobilization profile was seen in CD8^+^ T cells from relapsing patients when compared to HS (Table 3; Figure 2 and Supplementary Figure 3). This may due to the fact that many TCRVβ families have been differently mobilized in each clinical phase for each patient, suggesting that the CD8^+^ TCRVβ repertoire in R-VL/HIV patients is more heterogeneous, and may be related to a reduced ability of parasite control.

Again, the heatmap analysis of the CD8^+^ TCRVβ repertoire (Figure 3B) reinforced our previous observations, such as the high use of Vβ18 by 3 out of 5 NR-VL/HIV patients and only one out of 8 R-VL/HIV cases (Figure 3B) during the active VL. This family reduced in all NR-VL/HIV patients at 6 and 12 mpt. The Vβ23 family mobilization was also expressively reduced in all NR-VL/HIV patients at 12 mpt, with a trend to be more used among R-VL/HIV patients mainly in the VL active phase (4 out of 8). Finally, the levels of Vβ3 family were not only elevated during all the clinical follow-up of the NR-VL/HIV (3 out of 5 patients), but also tended to be more expressed in this group in comparison to R-VL/HIV patients (Figures 2A, 3B). Overall, an extremely heterogeneous pattern of CD8^+^ TCRVβ repertoire in the R-VL/HIV cases was detected, with numerous differences in the individual mobilization profile.

### Decrease of Pro-inflammatory Cytokine Levels in Non-relapsing VL/HIV Co-infected Patients

**Figure 4:**
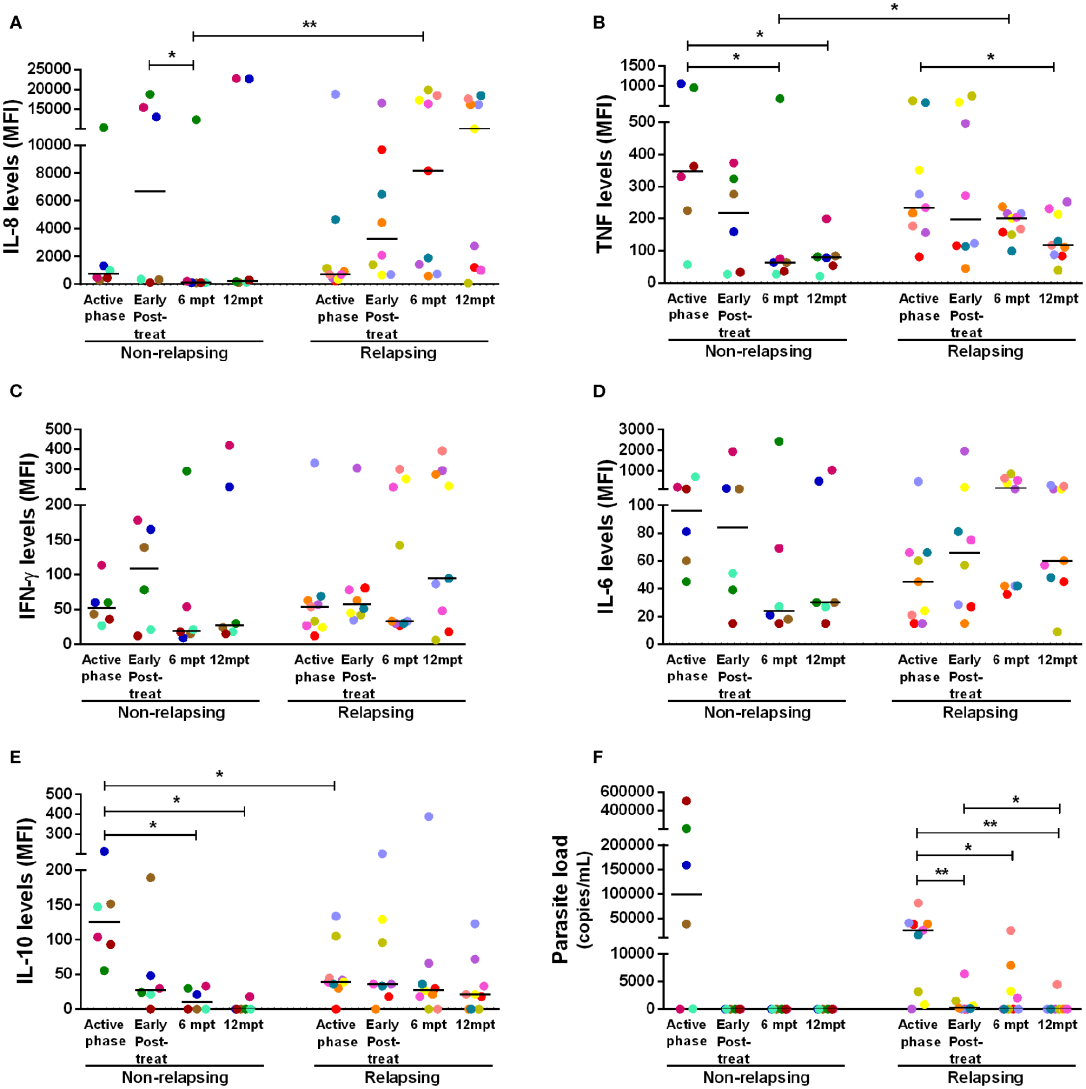
Anti- and pro-inflammatory cytokines levels and parasite load of the non-relapsing and relapsing-visceral leishmaniasis/HIV (VL/HIV) co-infected patients during clinical follow-up. Plasma cytokine levels of IL-8 **(A)**, TNF **(B)**, IFN-γ **(C)**, IL-6 **(D)**, IL-10 **(E)** and the parasite load **(F)** in NR-VL/HIV and R-VL/HIV co-infected patients in the active phase, early post-treatment, 6 and 12 mpt. The cytokines results were represented in Median Fluorescence Intensity (MFI). The cytokine levels were assessed by Luminex assay and the parasite load quantification by qPCR. Each point represents a VL/HIV co-infected patient and each color represents the same patient in the different phases of the follow-up. The horizontal bar represents the median values. Early Post-treat (early post-treatment); 6 mpt (6 months post-treatment); 12 mpt (12 months post-treatment). *p < 0.05 **p < 0.005.

Considering that NR-VL/HIV and R-VL/HIV patients differed in their T cell reconstitution profile (CD4^+^ T cell counts and TCRVβ repertoire), especially after 6 mpt, we investigated its potential impact upon the pro- and anti-inflammatory cytokine status of these patients. During the active phase and early post-treatment, NR-VL/HIV and R-VL/HIV patients exhibited similar levels of IL-8 and TNF (Figures 4A,B). Nevertheless, in NR-VL/HIV patients there was a significant decrease in the levels of these cytokines, from 6 mpt compared to the early phases. By contrast, R-VL/HIV patients kept or even augmented IL-8 and TNF levels, which were significantly higher than those observed in NR-VL/HIV cases (Figures 4A,B).

Interestingly, we also observed a significant decrease in IL-10 serum levels, in NR-VL/HIV patients at 6 and 12 mpt, in comparison to the VL active phase (Figure 4E). This finding may be associated to the fact of the parasite load in this group have become undetectable after anti-*Leishmania* treatment (Figure 4F). Moreover, we found a negative correlation between the IL-10 levels in the active phase of VL and the total number of VL episodes (*r* = −0.65, *p* < 0.05) (see Supplementary Figure 4H).

For the other cytokines, the same pattern was observed: the majority of the NR-VL/HIV patients showed a tendency to reduce IFN-γ and IL-6 (Figures 4C,D), as well as IL-2, IL-17, CCL4, IL-13, and IL-4 levels (see Supplementary Figures 4B,C,E–G) at 6 and 12 mpt in relation to the active phase and immediately after the anti-*Leishmania* treatment. On the other hand, among the R-VL/HIV patients, IFN-γ and IL-6 (Figures 4C,D), as well IL-1β, CCL2, CCL4, and IL-4 (see Supplementary Figures 4A,D,E,G) levels tended to remain elevated or even higher at 6 and 12 mpt in comparison to those found in the early periods of clinical follow-up.

### Thymic Output May Contribute to the Replenishment of T Cells in Non-relapsing VL/HIV Patients

**Figure 5:**
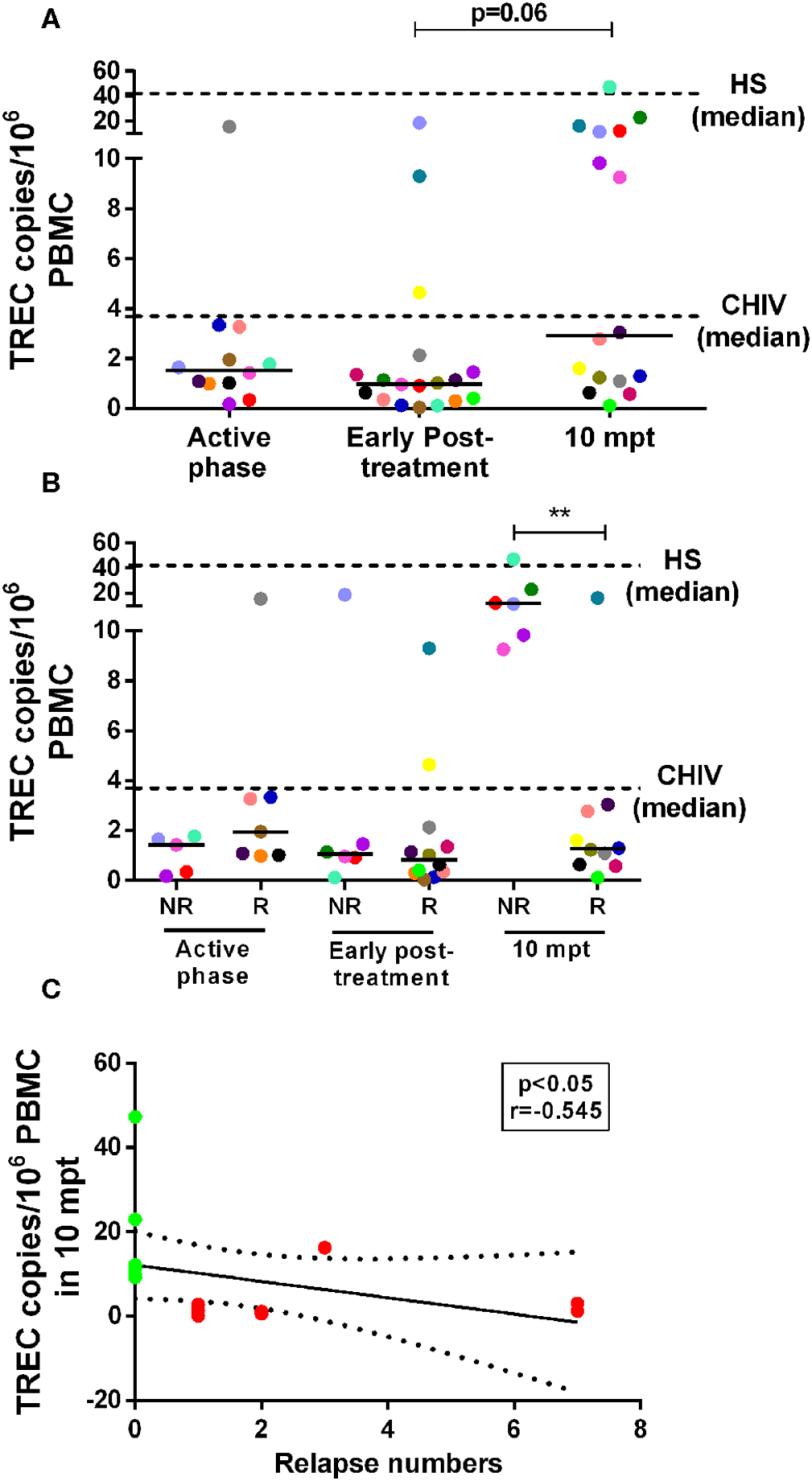
Number of T cell receptor excision circles (TREC) copies/10^6^ PBMC in visceral leishmaniasis/HIV (VL/HIV) co-infected patients during the prospective follow-up and its correlation with number of VL relapses. TREC copy numbers in the VL/HIV-co-infected patients **(A)** and TRECS copy numbers in non-relapsing-VL/HIV (NR-) and relapsing-VL/HIV (R-) group **(B)** during the clinical follow-up. The number of TREC copies was evaluated from 1 to 5 × 10^6^ cells/mL obtained of the peripheral blood of all VL/HIV co-infected patients in the active, early post-treatment and 10 mpt phases, as well as HIV mono-infected patients (CHIV) and healthy subjects (HS). Negative correlation **(C)** between the number of TREC copies at 10 mpt and the total number of relapses presented by VL/HIV patients (Spearman correlation, *r* = −0.545; *p* < 0.05). The green and red colors represent the NR-VL/HIV and R- patients, respectively **(C)**. The dashed lines represent the median of TREC copies/10^6^ PBMC of the HIV mono-infected patients and HS **(A,B)**. Each point represents a patient and each color represents the same patient in the different phases of follow-up. The horizontal bar represents the median values. 10 mpt (10 months post-treatment). **p < 0.005.

As we previously found that both NR-VL/HIV and R-VL/HIV individuals exhibit elevated percentages of senescent T cells (13), but different degree of immune reconstitution, inflammatory profile and mobilization of Vβ families, we questioned whether the thymic compartment could be contributing to this differential immune status. As depicted in Figure 5A, the numbers of TREC copies per million of PBMCs were lower in the VL/HIV co-infected patients during active phase (1.54 TRECs; IQR: 0.99–2.94 TRECs) and early post-treatment (0.99 TRECs; IQR: 0.34–1.62 TRECs) when compared to HIV mono-infected or healthy subjects (HIV: 3.71 TRECs; IQR: 1.74–17.37 TRECs; and HS: 42.08 TRECs; IQR: 15.41–116.6 TRECs). Nevertheless, a tendency to increase (p=0.06) was observed among co-infected patients at 10 mpt (2.91 TRECs; IQR: 1.13–11.99), as compared to the initial phases of the follow-up (Figure 5A). When these patients were split into relapsing (R-VL/HIV) and non-relapsing (NR-VL/HIV), it was observed that the augment in TRECs at 10 mpt was due to a significant increase in NR-VL/HIV patients in comparison to R-VL/HIV (Figure 5B). The median of TREC copies in PBMCs seen in NR-VL/HIV patients were higher than those observed in the HIV mono-infected individuals and some of NR-VL/HIV patients showed data rather close to the median of HS (Figure 5B, dashed line). By contrast, R-VL/HIV patients maintained low TREC copies during all the follow-up period, and below the medians of the control group (Figure 5B).

The significant increase in the numbers of TREC copies in NR-VL/HIV patients at 10 mpt (Figure 5B) may reflect their ability to replenish the pool of peripheral CD4^+^ T cells, as we showed in the Table 1.

Importantly, the TREC copy numbers per million PBMCs at 10 mpt negatively correlated (*p* < 0.05, *r* = −0.545) with the number of relapses observed in VL/HIV patients in this same period of follow-up, as seen in Figure 5C, again suggesting that the impairment of the thymic output may influence the occurrence of VL relapses.

## Discussion

Relapses in VL/HIV patients are a challenge for clinicians since it is, not only a frequent event, but also difficult to manage because of the scarce therapeutic options. A negative correlation between CD4^+^ T cell counts and cellular activation levels in VL/HIV co-infected patients was previously demonstrated (13). Corroborating with these results, relapsing-VL/HIV patients whose activation levels were elevated during 12 months of the clinical follow-up also maintained low CD4^+^ T-lymphocyte counts. On the other hand, non-relapsing-VL/HIV presented a gain of CD4^+^ T cells, but not of CD8^+^ T cells. Therefore, we decided to investigate the quality of the T cells circulating in the periphery, in terms of diversity and mobilization profile of Vβ families, cytokine status, and the probable origin of T cells.

Disturbances in the TCRVβ repertoire diversity are observed in HIV monoinfected patients at the acute phase of the infection (28, 29, 31, 38), perpetuating through chronic infection (31). Herein, we showed that VL/HIV co-infected patients also suffered significant disturbances in the expansion or retraction of several TCRVβ-families, mainly during active and VL post-treatment phases, regardless of these are relapsing or non-relapsing. In terms of TCRVβ on CD4^+^ T cells, NR-VL/HIV and R-VL/HIV groups mobilized different Vβ families when compared to healthy individuals. However, to the TCRVβ on CD8^+^ T cells it is interesting to note that the NR-VL/HIV patients mobilized several families either to a greater or lesser expression, whereas R-VL/HIV group mobilized few families; most of them being down expressed in comparison to the HS.

In this context, the analysis of these disturbances by heatmap suggested a more homogeneous TCRVβ repertoire on CD4^+^ T-cells, especially in the NR-VL/HIV cases. Differently, the TCRVβ repertoire on CD8^+^ T-cells is extremely heterogeneous when assessed individually, since different Vβ-families were mobilized, through expansions or retractions. These differential alterations were more intense among R-VL/HIV patients. Indeed, in HIV-1 infection, major disturbances have been described in the CD8^+^ TCRVβ repertoire, regardless of the clinical status, CD8^+^ and CD4^+^ T-cell counts, as well as viral load (39). On the other hand, the CD4^+^ TCRVβ repertoire appears to be severely disturbed when there are low CD4^+^ T cells counts and high HIV viremia (39). Considering that CD8^+^ T-cells are an important subpopulation for parasite control, we could infer that the intense disturbance on the CD8^+^ T-cell repertoire may be related to the predisposition to VL relapses.

Although a characteristic TCRVβ mobilization profile was not seen in neither R-VL/HIV nor NR-VL/HIV cases, it is noteworthy the fact that three Vβ families were significantly retracted at 12 mpt of the NR-VL/HIV patients in relation to the early stages of the follow-up (Vβ9 on CD4^+^ T-cells; Vβ18 on CD8^+^ T-cells; Vβ23 in both CD8^+^ and CD4^+^ T-cells, *p* < 0.05). Of these, only two (Vβ9 on CD4^+^ T-cells; Vβ18 on CD8^+^) were highly mobilized in the active phase of this group. Similarly, R-VL/HIV patients also presented a reduction in the mobilization of some Vβ families (Vβ9 and Vβ18 on CD4^+^ and Vβ23 on CD8^+^ T cells, *p* < 0.05) in relation to the active phase. Moreover, the Vβ3 family was less mobilized in CD4^+^ and CD8^+^ T-cells in R-VL/HIV patients throughout the clinical follow-up, although it is a Vβ family that is usually increased in Brazilian HIV-positive patients, with or without ART (29). These retractions, regardless of whether it is R or NR, could be related to the return to baseline state of expression of Vβ families or even to a severe clonal exhaustion process. Considering the continuous *Leishmania* stimulation in the scenario of VL/HIV co-infection, it is plausible that the exhaustion of primary immune resources may influence the effector immune response and therefore the occurrence of relapse episodes. Herein, we showed that there are changes in the TCRVβ mobilization profile of co-infected patients, especially during the clinical follow-up. Even so, the association between the mobilization of these families and parasitic control could not be addressed in this study. New approaches based on other study designs and using specific stimulations may clarify these questions.

It is important to stress that cytofluorometry is not informative for evaluating the specificity of a given TCR repertoire or defining clonotypes. Herein, this strategy aimed at determining if the percentages of T cells using a given Vβ family differed between patients and controls and for evaluating the dynamics of mobilizations in terms of peripheral frequency, especially after an immunological reconstitution.

In addition to the gain in CD4^+^T lymphocyte numbers, the reduction of the T-cell activation status was markedly observed in VL/HIV patients who achieved clinical remission after anti-leishmanial therapy (13). Then, a possible relationship between the improvement of the immune status along with the replenishment of T lymphocytes pool (CD4^+^ T cell gain and TCRVβ repertoire) on the profile of systemic inflammatory cytokine, was addressed. We found a tendency of the NR-VL/HIV group to reduce the plasmatic pro- and anti-inflammatory cytokine levels throughout the clinical follow-up. In this respect, low levels of IL-10 in the active phase of the disease in R-VL/HIV group may contribute to the maintenance of an activated and inflammatory immune status, due to the lack of regulatory action of this cytokine, as suggested by negative correlation observed between this parameter and the VL relapse numbers. A cytokine storm has been associated with the severity of VL alone (9), with the worsening of the immune status and progression to AIDS in HIV-positive individuals (40). Moreover, it has been associated with the presence of *Leishmania* in VL/HIV patients (7). As in a vicious circle, this inflammatory status may contribute to maintaining the high levels of cellular activation that, in turn, continuously compromises the general immune status of relapsing-VL/HIV patients, generating exhaustion and peripheral senescence, thus compromising central immune functions.

Despite the reduction of inflammatory status in NR-VL/HIV patients, the high proportion of peripheral senescent T cells previously verified (13) indicates that these patients present an impairment of the T cell proliferative capacity. This raised the question on the origin of the newly-formed T cells detected in the circulation of NR-VL/HIV patients, which prompted us to investigate a putative thymus participation in this process. Herein, for co-infected patients, the TREC copy numbers were low during the VL active phase, suggesting a thymic functional impairment in the renewal of the T-cell pool, despite ART use and low or undetectable viral load in the majority of patients (13). In fact, it is well-described that thymic functionality is compromised in HIV-infected patients, especially in those without ART (20, 21, 32, 41, 42), which is in keeping with the fact that the virus has the ability to infect or at least to affect thymic stromal cells, T-lymphocyte progenitors and thymocytes, resulting in lower production of new T-cells with consequent impairment of the immune reconstitution (43–45). However, TREC copies in VL/HIV co-infected patients were even lower than those seen in HIV-solely infected patients. This fact points out that not only residual HIV, but also *Leishmania* can be contributing to the severely impaired thymic function in VL/HIV patients. Previous studies have shown that *Leishmania* can affect T-cell progenitors in the bone marrow and the thymic microenvironment (46–48), which may also favor the deficient thymic output under conditions of VL. Additionally, as previously described, the parasite potentiates the immune activation degree (5, 49), mainly in R-VL/HIV cases (13), which in turn may lead to thymic dysfunction.

It is noteworthy that such thymic output deficiency seems to be more severe in those patients presenting VL relapses, since they maintained low TREC levels during all clinical setpoints of the follow-up, whereas non-relapsing-VL/HIV patients recovered these values at 10 mpt. Considering that TREC copy numbers were accompanied by maintenance of low absolute CD4^+^ T-cell counts in relapsing-VL/HIV and correlated negatively with the number of relapses, we suggest that the thymic impairment favors the loss of parasitic control and the recurrences in visceral leishmaniasis. Future quantification of TREC copies in sorted CD4^+^ and CD8^+^ T-cells would be useful to define if the CD4^+^ T-cell counts recovery among non-relapsing cases is related to increased TREC copy numbers in this subpopulation.

To the best of our knowledge no other study has reported an evaluation of thymic output in VL/HIV co-infected patients. In this scenario, along with TCRVβ repertoire disturbances and intense inflammatory status, it is expected that relapsing-VL/HIV patients present a qualitative deficit in the effector cellular immune response, which in turn may predispose to VL relapses. This set of factors may culminate in a higher susceptibility to VL relapses among VL/HIV co-infected patients who have a deficient immune system *per se* and that, along with each relapse, become increasingly unable to control the parasite.

In conclusion, our findings indicate that impaired thymic output is related to the low immune reconstitution and T cell repertoire disturbances in relapsing visceral leishmaniasis associated HIV/AIDS patients.

## Data Availability Statement

All datasets generated for this study are included in the article/Supplementary Material.

## Ethics Statement

The studies involving human participants were reviewed and approved by Hospital Eduardo de Menezes and Oswaldo Cruz Foundation (René Rachou and Oswaldo Cruz Institutes). The patients/participants provided their written informed consent to participate in this study.

## Author Contributions

MS-F, GC-C, JS-O, and AD-C: formal analysis, investigation, methodology, organized the database, and wrote the draft of the manuscript. JS-O and AD-C: conceptualization, funding acquisition and project administration. WS: formal analysis and critically revised the manuscript for intellectual content. GC: recruitment and clinical follow-up of the patients. JT and ZV: methodology and organized the database of TREC. CG-G and AR: writing— review and editing of the manuscript. All authors read and approved the final manuscript.

## Conflict of Interest

The authors declare that the research was conducted in the absence of any commercial or financial relationships that could be construed as a potential conflict of interest.

## Supplementary Material

Supplementary Figure 1 Percentages of T-cell receptor Vβ families among non-relapsing (NR) and relapsing (R) visceral leishmaniasis/HIV (VL/HIV) co-infected patients during the VL active phase and 12 months post treatment. Mobilization levels of the 24 Vβ families in NR-VL/HIV and R-VL/HIV co-infected patients in the active phase **(A,C)** and 12 mpt **(B,D)**. This evaluation was performed in accordance with the literature to indicate the families that were less (<1%) and more (>5%) mobilized by CD4^+^ **(A,B)** and CD8^+^ **(C,D)** T-cells Vβ repertoire. These mobilization limits (1% and 5%) were represented by dashed lines. The asterisks point to the significant differences for higher (blue) and lower (red) mobilization in the VL/HIV groups in relation to healthy subjects (HS). The column bar represents the median values with interquartile range. 12 mpt (12 months post-treatment). ^*^*p* < 0.05.

Supplementary Figure 2 Mobilization profile of CD4^+^ TCRVβ repertoire presented by non-relapsing (NR) and relapsing (R) visceral leishmaniasis/HIV (VL/HIV) co-infected patients during clinical follow-up. The percentages of the 19 out of 24 Vβ families that makes up the CD4^+^ T-cell repertoire were evaluated in the VL/HIV co-infected patients during clinical follow-up and in healthy subjects (HS). The column bar represents the median values with interquartile range. The blue asterisk represents the difference statistical in relation to HS. ^*^*p* < 0.05.

Supplementary Figure 3 Mobilization profile of CD8^+^ TCRVβ repertoire presented by non-relapsing (NR) and relapsing (R) visceral leishmaniasis/HIV (VL/HIV) co-infected patients during clinical follow-up. The percentages of the 19 out of 24 Vβ families that makes up the CD8^+^ T-cell repertoire were evaluated in the VL/HIV co-infected patients during clinical follow-up and in healthy subjects (HS). The blue asterisk represents the difference statistical in relation to HS. The column bar represents the median values with interquartile range. ^*^*p* < 0.05, ^**^*p* < 0.005.

Supplementary Figure 4 Anti- and pro-inflammatory cytokines and chemokines levels of the visceral leishmaniasis/HIV (VL/HIV) co-infected patients during clinical follow-up. Levels of IL-1β **(A)**, IL-2 **(B)**, IL-17 **(C)**, CCL2 (MCP-1) **(D)**, CCL4 (MIP-1β) **(E)**, IL-13 **(F)**, and IL-4 **(G)** in NR-VL/HIV and R-VL/HIV co-infected patients throughout clinical follow-up. **(H)** Negative correlation between the IL-10 levels during VL active phase and the total number of VL episodes in the VL/HIV patients (NR- and R- groups). The measure of these soluble factors was performed by Luminex assay. The results were represented as Median Fluorescence Intensity (MFI). Each point represents a VL/HIV co-infected patient and each color represents the same patient in the different phases of the follow-up. The horizontal bar represents the median values. Early Post-treat (early post-treatment); 6 mpt (6 months post-treatment); 12 mpt (12 months post-treatment). ^*^*p* < 0.05 ^**^*p* < 0.005.

## References

[1] Pagliano P Esposito S. Visceral leishmaniosis in immunocompromised host: an update and literature review. J Chemother. (2017) 29:261–6. 10.1080/1120009X.2017.1323150 28490252

[2] Lindoso JAL Moreira CHV Cunha MA Queiroz IT Visceral leishmaniasis and HIV co-infection: current perspectives. HIV/AIDS Res Palliat Care. (2018) 10:193–201. 10.2147/HIV.S143929 PMC6197215 30410407

[3] Ministério da Saúde Situação Epidemiológica da Leishmaniose Visceral. Available online at: http://portalarquivos2.saude.gov.br/images/pdf/2019/janeiro/28/leishvisceral-17-novo-layout.pdf%0Afile:///D:/IC/Pibic/leishvisceral-17-novo-layout.pdf (accessed August 1, 2019).

[4] Fontoura IG Barbosa DS de Andrade Paes AM Santos FS Neto MS Fontoura VM Epidemiological, clinical and laboratory aspects of human visceral leishmaniasis (HVL) associated with human immunodeficiency virus (HIV) co-infection: a systematic review. Parasitology. (2018) 145:1819 10.1017/S0031182018001166 30079849

[5] Santos-Oliveira JR Giacoia-Gripp CBW Alexandrino de Oliveira P Amato VS Lindoso JAL Goto H. High levels of T lymphocyte activation in *Leishmania*-HIV-1 co-infected individuals despite low HIV viral load. BMC Infect Dis. (2010) 10:358. 10.1186/1471-2334-10-358 21171992 PMC3022832

[6] Santos-Oliveira JR Regis EG Leal CRB Cunha RV Bozza PT Da-Cruz AM. Evidence that lipopolisaccharide may contribute to the cytokine storm and cellular activation in patients with visceral leishmaniasis. PLoS Negl Trop Dis. (2011) 5:e1198. 10.1371/journal.pntd.0001198 21765960 PMC3134430

[7] Santos-Oliveira JR Regis EG Giacoia-Gripp CBW Valverde JG Alexandrino-De-Oliveira P Lindoso JAL Microbial translocation induces an intense proinflammatory response in patients with visceral leishmaniasis and HIV type 1 co-infection. J Infect Dis. (2013) 208:57–66. 10.1093/infdis/jit135 23539743

[8] Costa CHN Werneck GL Costa DL Holanda TA Aguiar GB Carvalho AS. Is severe visceral leishmaniasis a systemic inflammatory response syndrome? A case control study. Rev Soc Bras Med Trop. (2010) 43:386–92. 10.1590/s0037-86822010000400010 20802936

[9] Costa DL Rocha RL Carvalho RMA Lima-Neto AS Harhay MO Costa CHN. Serum cytokines associated with severity and complications of kala-azar. Pathog Glob Health. (2013) 107:78–87. 10.1179/2047773213Y.0000000078 23683334 PMC4001482

[10] Dayakar A Chandrasekaran S Kuchipudi S V Kalangi SK. Cytokines: key determinants of resistance or disease progression in visceral leishmaniasis: opportunities for novel diagnostics and immunotherapy. Front Immunol. (2019) 10:670. 10.3389/fimmu.2019.00670 31024534 PMC6459942

[11] Silva RLL Santos MB Almeida PLS Barros TS Magalhães L Cazzaniga RA. sCD163 levels as a biomarker of disease severity in leprosy and visceral leishmaniasis. PLoS Negl Trop Dis. (2017) 11:e0005486. 10.1371/journal.pntd.0005486 28355218 PMC5386291

[12] Cunha Fievez da AM Silva-Freitas ML Sousa AQ Santos-Oliveira JR Da-Cruz AM Lower levels of leptin are associated with severity parameters in visceral leishmaniasis patients. PLoS ONE. (2019) 14:e0214413 10.1371/journal.pone.0214413 30913261 PMC6435192

[13] Silva-Freitas ML Cota GF Machado-De-Assis TS Giacoia-Gripp C Rabello A Da-Cruz AM. Immune activation and bacterial translocation: a link between impaired immune recovery and frequent visceral leishmaniasis relapses in HIV-infected patients. PLoS ONE. (2016) 11:e0167512. 10.1371/journal.pone.0167512 27907136 PMC5132299

[14] Cota GF de Sousa MR de Assis TSM Pinto BF Rabello A. Exploring prognosis in chronic relapsing visceral leishmaniasis among HIV-infected patients: circulating *Leishmania* DNA. Acta Trop. (2017) 172:186–91. 10.1016/j.actatropica.2017.05.011 28501450

[15] Douek DC Koup RA McFarland RD Sullivan JL Luzuriaga K. Effect of HIV on thymic function before and after antiretroviral therapy in children. J Infect Dis. (2000) 181:1479–82. 10.1086/315398 10762580

[16] Appay V Almeida JR Sauce D Autran B Papagno L. Accelerated immune senescence and HIV-1 infection. Exp Gerontol. (2007) 42:432–7. 10.1016/j.exger.2006.12.003 17307327

[17] Sokoya T Steel HC Nieuwoudt M Rossouw TM. HIV as a cause of immune activation and immunosenescence. Mediators Inflamm. (2017) 2017:6825493. 10.1155/2017/6825493 29209103 PMC5676471

[18] Savino W. The thymus is a common target organ in infectious diseases. PLoS Pathog. (2006) 2:e62. 10.1371/journal.ppat.0020062 16846255 PMC1483230

[19] Douek DC McFarland RD Keiser PH Gage EA Massey JM Haynes BF. Changes in thymic function with age and during the treatment of HIV infection. Nature. (1998) 396:690–5. 10.1038/25374 9872319

[20] Ferrando-Martinez S De Pablo-Bernal RS De Luna-Romero M De Ory SJ Genebat M Pacheco YM. Thymic function failure is associated with human immunodeficiency virus disease progression. Clin Infect Dis. (2017) 64:1191–7. 10.1093/cid/cix095 28158588 PMC6248450

[21] Rb-Silva R Nobrega C Azevedo C Athayde E Canto-Gomes J Ferreira I. Thymic function as a predictor of immune recovery in chronically HIV-infected patients initiating antiretroviral therapy. Front Immunol. (2019) 10:25. 10.3389/fimmu.2019.00025 30804925 PMC6370619

[22] Salameire D Solly F Fabre B Lefebvre C Chauvet M Gressin R Accurate detection of the tumor clone in peripheral T-cell lymphoma biopsies by flow cytometric analysis of TCR-VB repertoire. Mod Pathol. (2012) 25:1246–57. 10.1038/modpathol.2012.74 22627740

[23] Chang CM Hsu YW Wong HSC Wei JCC Liu X Liao HT. Characterization of T-cell receptor repertoire in patients with rheumatoid arthritis receiving biologic therapies. Dis Markers. (2019) 2019:2364943. 10.1155/2019/2364943 31360262 PMC6642763

[24] Plasilova M Risitano A Maciejewski JP. Application of the molecular analysis of the T-cell receptor repertoire in the study of immune-mediated hematologic diseases. Hematology. (2003) 8:173–81. 10.1080/1024533031000107505 12745651

[25] Costa RP Gollob KJ Fonseca LL Rocha MOC Chaves ACL Medrano MN T-cell repertoire analysis in acute and chronic human Chagas'disease: differential frequencies of Vβ5 expressing T cells. Scand J Immunol. (2000) 51:511–9. 10.1046/j.1365-3083.2000.00706.x 10792844

[26] Ferraz R Cunha CF Pimentel MI Lyra MR Schubach AO Mendonça SCF. T-cell receptor Vβ repertoire of CD8^+^ T-lymphocyte subpopulations in cutaneous leishmaniasis patients from the state of Rio de Janeiro, Brazil. Mem Inst Oswaldo Cruz. (2015) 110:596–605. 10.1590/0074-02760150039 26107186 PMC4569821

[27] Clarencio J de Oliveira CI Bomfim G Pompeu MM Teixeira MJ Barbosa TC. Characterization of the T-cell receptor Vbeta repertoire in the human immune response against *Leishmania* parasites. Infect Immun. (2006) 74:4757–65. 10.1128/IAI.00265-06 16861664 PMC1539606

[28] Pantaleo G Demarest JF Soudeyns H Graziosi C Denis F Adelsberger JW Major expansion of CD8^+^ T cells with a predominant Vbeta usage during the primary immune response to HIV. Nature. (1994) 370:463–7. 10.1038/370463a0 8047166

[29] Giacoia-Gripp CBW Neves I Galhardo MC Morgado MG. Flow cytometry evaluation of the T-cell receptor Vbeta repertoire among HIV-1 infected individuals before and after antiretroviral therapy. J Clin Immunol. (2005) 25:116–26. 10.1007/s10875-005-2817-z 15821888

[30] Hernández DM Valderrama S Gualtero S Hernández C López M Herrera MV. Loss of T-cell multifunctionality and TCR-Vβ repertoire against Epstein-Barr virus is associated with worse prognosis and clinical parameters in HIV^+^ patients. Front Immunol. (2018) 9:2291. 10.3389/fimmu.2018.02291 30337929 PMC6180205

[31] Heather JM Best K Oakes T Gray ER Roe JK Thomas N. Dynamic perturbations of the T-cell receptor repertoire in chronic HIV infection and following antiretroviral therapy. Front Immunol. (2016) 6:644. 10.3389/fimmu.2015.00644 26793190 PMC4707277

[32] Zicari S Sessa L Cotugno N Ruggiero A Morrocchi E Concato C Immune activation, inflammation, and non-AIDS comorbidities in HIV-infected patients under long-term ART. Viruses. (2019) 11:200 10.3390/v11030200 PMC6466530 30818749

[33] Furler RL Newcombe KL Del Rio Estrada PM Reyes-Terán G Uittenbogaart CH Nixon DF. Histoarchitectural deterioration of lymphoid tissues in HIV-1 infection and in aging. AIDS Res Hum Retroviruses. (2019) 35:1148–59. 10.1089/AID.2019.0156 31474115 PMC6862967

[34] Losada-Barragán M Umaña-Pérez A Cuervo-Escobar S Berbert LR Porrozzi R Morgado FN Protein malnutrition promotes dysregulation of molecules involved in T cell migration in the thymus of mice infected with *Leishmania infantum*. Sci Rep. (2017) 7:45991 10.1038/srep45991 28397794 PMC5387407

[35] Ministério da Saúde *Manual de recomendações para diagnóstico, tratamento e acompanhamento de pacientes com a coinfecção Leishmania*-HIV. 2011. Available online at: http://bvsms.saude.gov.br/bvs/publicacoes/manual_recomendacoes_pacientes_leishmania.pdf (accessed September 1, 2019).

[36] Cota GF de Sousa MR de Mendonça ALP Patrocinio A Assunção LS de Faria SR. *Leishmania*-HIV co-infection: clinical presentation and outcomes in an urban area in Brazil. PLoS Negl Trop Dis. (2014) 8:e2816. 10.1371/journal.pntd.0002816 24743472 PMC3990491

[37] Breen EJ Polaskova V Khan A. Bead-based multiplex immuno-assays for cytokines, chemokines, growth factors and other analytes: median fluorescence intensities versus their derived absolute concentration values for statistical analysis. Cytokine. (2015) 71:188–98. 10.1016/j.cyto.2014.10.030 25461398

[38] Yin L Zhong CK Rodriguez C Hou W Goodenow MM Sleasman JW. Antiretroviral therapy restores diversity in the T-cell receptor Vβ repertoire of CD4 T-cell subpopulations among human immunodeficiency virus type 1-infected children and adolescents. Clin Vaccine Immunol. (2009) 16:1293–301. 10.1128/CVI.00074-09 19605599 PMC2745006

[39] Gorochov G Neumann AU Kereveur A Parizot C Li T Katlama C. Perturbation of CD4^+^ and CD8^+^ T-cell repertoires during progression to AIDS and regulation of the CD4^+^ repertoire during antiviral therapy. Nat Med. (1998) 4:215–21. 10.1038/nm0298-215 9461196

[40] Leeansyah E Malone DFG Anthony DD Sandberg JK. Soluble biomarkers of HIV transmission, disease progression and comorbidities. Curr Opin HIV AIDS. (2013) 8:117–24. 10.1097/COH.0b013e32835c7134 23274365

[41] Rosado-Sánchez I Herrero-Fernández I Genebat M Ruiz-Mateos E Leal M Pacheco YM. Thymic function impacts the peripheral CD4/CD8 ratio of HIV-infected subjects. Clin Infect Dis. (2017) 64:152–8. 10.1093/cid/ciw711 27986677

[42] De Voeght A Martens H Renard C Vaira D Debruche M Simonet J. Exploring the link between innate immune activation and thymic function by measuring sCD14 and TRECs in HIV patients living in Belgium. PLoS ONE. (2017) 12:e185761. 10.1371/journal.pone.0185761 29049344 PMC5648129

[43] Savino W Dardenne M Marche C Trophilme D Dupuy JM Pekovic D. Thymic epithelium in AIDS. An immunohystologic study. Am J Pathol. (1986) 122:302–7. 3511724 PMC1888096

[44] Ye P Kirschner D Kourtis A. The thymus during HIV disease: role in pathogenesis and in immune recovery. Curr HIV Res. (2004) 2:177–83. 10.2174/1570162043484898 15078181

[45] Savino W Mendes-da-Cruz DA Lepletier A Dardenne M. Hormonal control of T-cell development in health and disease. Nat Rev Endocrinol. (2016) 12:77–89. 10.1038/nrendo.2015.168 26437623

[46] Cuervo-Escobar S Losada-Barragán M Umaña-Pérez A Porrozzi R Saboia-Vahia L Miranda LHM. T-cell populations and cytokine expression are impaired in thymus and spleen of protein malnourished BALB/c mice infected with *Leishmania infantum*. PLoS ONE. (2014) 9:e114584. 10.1371/journal.pone.0114584 25535967 PMC4275170

[47] Kumar P Misra P Thakur CP Saurabh A Rishi N Mitra DK. T cell suppression in the bone marrow of visceral leishmaniasis patients: impact of parasite load. Clin Exp Immunol. (2018) 191:318–27. 10.1111/cei.13074 29058314 PMC5801524

[48] Losada-Barragán M Umaña-Pérez A Durães J Cuervo-Escobar S Rodríguez-Vega A Ribeiro-Gomes FL. Thymic microenvironment is modified by malnutrition and *Leishmania infantum* infection. Front Cell Infect Microbiol. (2019) 9:252. 10.3389/fcimb.2019.00252 31355153 PMC6639785

[49] Casado JL Abad-Fernández M Moreno S Pérez-Elías MJ Moreno A Bernardino JI Visceral leishmaniasis as an independent cause of high immune activation, T-cell senescence, and lack of immune recovery in virologically suppressed HIV-1-co-infected patients. HIV Med. (2015) 16:240–8. 10.1111/hiv.12206 25604328

