# Supplementary figures and images for: Impaired Thymic Output Can Be Related to the Low Immune Reconstitution and T Cell Repertoire Disturbances in Relapsing Visceral Leishmaniasis Associated HIV/AIDS Patients
Source: Front Immunol. 2020 May 20;11:953. doi: 10.3389/fimmu.2020.00953 (PMC7251171; doi:10.3389/fimmu.2020.00953)

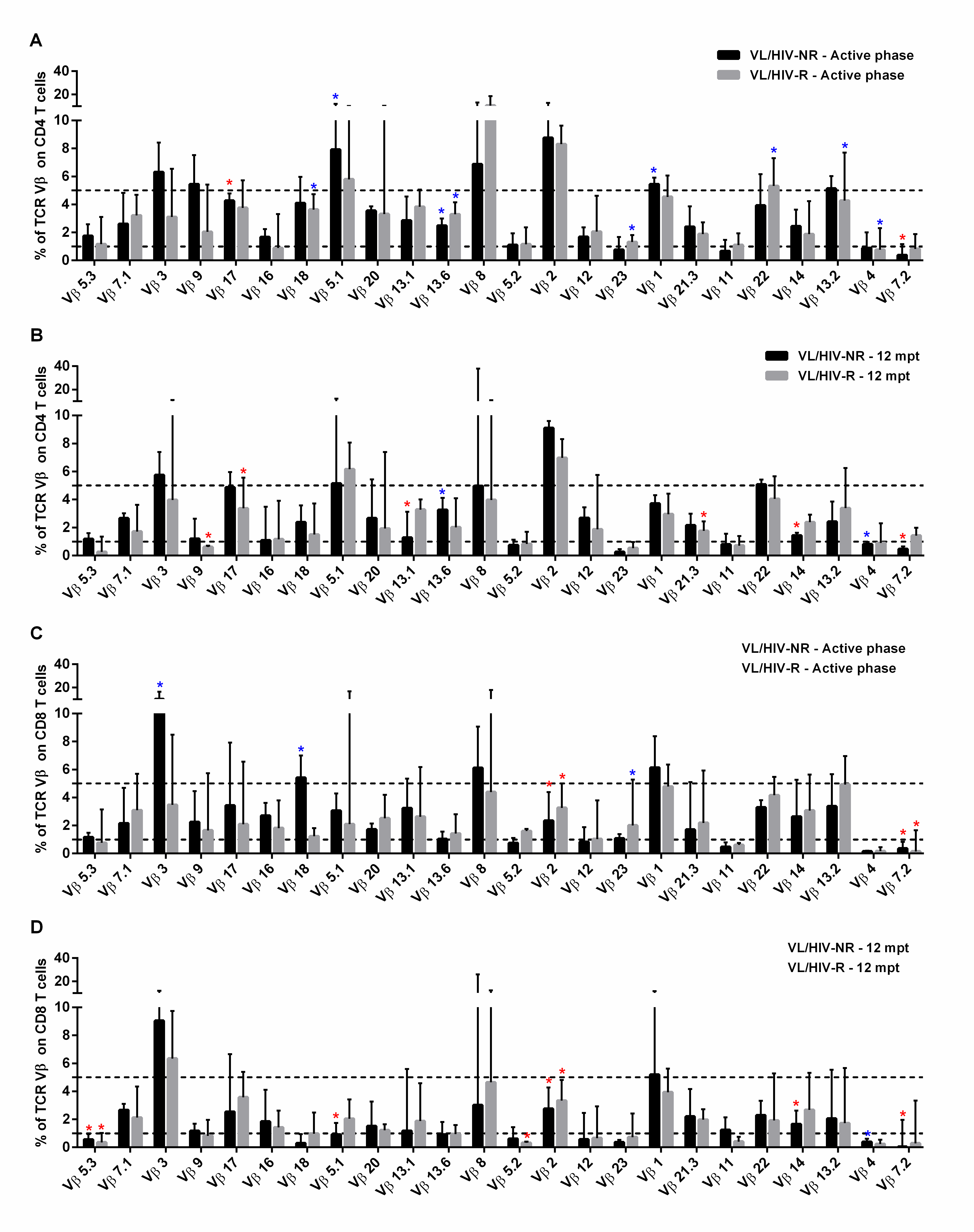

Supplement: Supplementary Figure 1 — Percentages of T-cell receptor Vβ families among non-relapsing (NR) and relapsing (R) visceral leishmaniasis/HIV (VL/HIV) co-infected patients during the VL active phase and 12 months post treatment. Mobilization levels of the 24 Vβ families in NR-VL/HIV and R-VL/HIV co-infected patients in the active phase (A,C) and 12 mpt (B,D). This evaluation was performed in accordance with the literature to indicate the families that were less (<1%) and more (>5%) mobilized by CD4+ (A,B) and CD8+ (C,D) T-cells Vβ repertoire. These mobilization limits (1% and 5%) were represented by dashed lines. The asterisks point to the significant differences for higher (blue) and lower (red) mobilization in the VL/HIV groups in relation to healthy subjects (HS). The column bar represents the median values with interquartile range. 12 mpt (12 months post-treatment). *p < 0.05. [file Image_1.TIF]
